# Paracrine IL-33 Stimulation Enhances Lipopolysaccharide-Mediated Macrophage Activation

**DOI:** 10.1371/journal.pone.0018404

**Published:** 2011-04-11

**Authors:** Tatsukuni Ohno, Keisuke Oboki, Hideaki Morita, Naoki Kajiwara, Ken Arae, Shizuko Tanaka, Masako Ikeda, Motoyasu Iikura, Taishin Akiyama, Jun-ichiro Inoue, Kenji Matsumoto, Katsuko Sudo, Miyuki Azuma, Ko Okumura, Thomas Kamradt, Hirohisa Saito, Susumu Nakae

**Affiliations:** 1 Department of Allergy and Immunology, National Research Institute for Child Health and Development, Tokyo, Japan; 2 Department of Molecular Immunology, Graduate School of Medical and Dental Science, Tokyo Medical and Dental University, Tokyo, Japan; 3 Atopy Research Center, Juntendo University, Tokyo, Japan; 4 Technical and Research Department, Ina Laboratory, Medical and Biological Laboratories Co., Ltd., Nagano, Japan; 5 Department of Respiratory Medicine, International Medical Center of Japan, Tokyo, Japan; 6 Division of Cellular and Molecular Biology, The Institute of Medical Science, The University of Tokyo, Tokyo, Japan; 7 Frontier Research Initiative, The Institute of Medical Science, The University of Tokyo, Tokyo, Japan; 8 Laboratory of Systems Biology, Center for Experimental Medicine and Systems Biology, The Institute of Medical Science, The University of Tokyo, Tokyo, Japan; 9 Animal Research Center, Tokyo Medical University, Tokyo, Japan; 10 Institut für Immunologie, Universitätsklinikum Jena, Jena, Germany; Centre d'Immunologie de Marseille-Luminy, CNRS-Inserm, France

## Abstract

**Background:**

IL-33, a member of the IL-1 family of cytokines, provokes Th2-type inflammation accompanied by accumulation of eosinophils through IL-33R, which consists of ST2 and IL-1RAcP. We previously demonstrated that macrophages produce IL-33 in response to LPS. Some immune responses were shown to differ between ST2-deficient mice and soluble ST2-Fc fusion protein-treated mice. Even in anti-ST2 antibody (Ab)-treated mice, the phenotypes differed between distinct Ab clones, because the characterization of such Abs (i.e., depletion, agonistic or blocking Abs) was unclear in some cases.

**Methodology/Principal Findings:**

To elucidate the precise role of IL-33, we newly generated neutralizing monoclonal Abs for IL-33. Exogenous IL-33 potentiated LPS-mediated cytokine production by macrophages. That LPS-mediated cytokine production by macrophages was suppressed by inhibition of endogenous IL-33 by the anti-IL-33 neutralizing mAbs.

**Conclusions/Significance:**

Our findings suggest that LPS-mediated macrophage activation is accelerated by macrophage-derived paracrine IL-33 stimulation.

## Introduction

IL-33 (also called IL-1F11, DVS27 and NF-HEV), which is a member of the IL-1 family of cytokines that includes IL-1 and IL-18, was identified as a ligand for ST2 (also called T1, DER-4, Fit-1 and IL-1R4) [1], [2], [3], [4]. IL-33 is considered to be a cytokine that potently induces production of such Th2-cytokines as IL-5 and IL-13 by ST2-expressing immune cells such as Th2 cells [1], [5], [6], mast cells [7], [8], [9], [10], [11], eosinophils [6], [12], [13], basophils [12], [13], [14] and macrophages [15], [16], and by stem-cell-like cells such as CD34^+^ hematopoietic stem cells [17], natural helper cells [18] and nuocytes [19]. IL-33 is thereby thought to contribute to the development of Th2-cytokine-associated immune responses, including host defense against nematode infection and allergic diseases [2], [3], [4].

Indeed, administration of IL-33 to mice resulted in increased serum levels of Th2-cytokines such as IL-4, IL-5 and IL-13, as well as IgG1 and IgE, and development of inflammation accompanied by accumulation of eosinophils in the lung and gut [1]. Moreover, polymorphism of the ST2 and/or IL-33 genes was found in patients with asthma [20], [21], [22], atopic dermatitis [23], rhinitis [24] and rhinosinusitis [25]. The mRNA and/or protein levels of ST2, soluble ST2, which acts as a decoy receptor for IL-33, and IL-33 are increased in specimens from patients with allergic diseases such as asthma [26], [27], [28], [29], [30], [31], conjunctivitis [31], rhinitis [24] and atopic dermatitis [32]. Therefore, these observations strongly suggest the importance of IL-33 and ST2 for the development of Th2-cytokine-associated allergic disorders.

However, based on the results of a study using mice treated with anti-ST2 Ab or soluble ST2-Fc fusion proteins and/or deficient in ST2, the roles of IL-33 and ST2 in the pathogenesis of certain immune diseases, including allergic airway inflammation, remain controversial [4]. Studies using ST2-deficient mice found that ovalbumin (OVA)-induced airway inflammation developed normally in ST2-deficient mice sensitized twice with OVA emulsified with alum [33], [34], [35], whereas it was attenuated in the case of a single sensitization [35]. On the other hand, mice treated with anti-ST2 mAb clone “3E10,” which induced Th2 cell activation as an agonistic Ab, at least *in vitro* [36], without depleting ST2-expressing cells *in vivo* [37], and mice treated with soluble ST2 showed reduced development of OVA-induced airway inflammation, even though they were sensitized twice with OVA with alum [38], [39]. Unlike in ST2-deficient mice [33], [34], [35], the development of OVA-induced airway inflammation was aggravated in mice injected with ST2-deficient OVA-specific TCR (DO11.10)-expressing Th2 cells in comparison with those injected with wild-type DO11.10 Th2 cells after OVA challenge [34]. That finding suggests that ST2 plays a negative role in Th2 cells, at least in that setting. On the other hand, it was shown that administration of anti-ST2 mAb “3E10” and soluble ST2-Fc fusion proteins to mice injected with DO11.10 Th2 cells resulted in attenuation of OVA-induced airway inflammation [38], [40]. These seemingly contradictory observations could be explained on the basis of different roles for IL-33 and ST2 in distinct ST2-expressing cells. In support of that concept, IL-33 is able to enhance IFN-γ production by NK cells and iNKT cells [26], which are also involved in the pathogenesis of allergic airway inflammation [41], [42]. Therefore, the precise roles of IL-33 and ST2 in different types of cells need to be elucidated.

We and others have demonstrated that IL-33 is able to enhance cytokine secretion by mast cells [7], [9] and macrophages [43]. We also reported that both mast cells and macrophages can produce IL-33 after stimulation with IgE and LPS, respectively [44]. These observations suggest that IL-33 may be involved in the activation of these cells by autocrine/paracrine IL-33 release after such stimulation. In the present study, we used newly generated anti-IL-33 mAbs and demonstrated that activation of macrophages, but not mast cells, was modulated by paracrine IL-33 stimulation.

## Materials and Methods

### Mice

BALB/cA (BALB) mice, C57BL/6J (B6J) mice and C57BL/6N (B6N) mice were purchased from CLEA Japan and Sankyo Lab, respectively. B6J-TLR4^−/−^ mice [45] and BALB-ST2^−/−^ mice [46] were kindly provided by Drs. Tsuneyasu Kaisho (RIKEN, Japan) and Andrew N.J. McKenzie (MRC, Cambridge, UK), respectively. B6J-TRAF6^−/−^ mice [47] and B6N-IL-33^−/−^ mice [48] were generated as described elsewhere. All mice were housed under specific-pathogen-free conditions in our institutes (National Research Institute for Child Health and Development or The Institute of Medical Science, The University of Tokyo), and the animal protocols were approved by the Institutional Review Board of the National Research Institute for Child Health and Development (#06-10) and The Institute of Medical Science, The University of Tokyo (#A09-10).

### Anti-mouse ST2 Abs

Anti-mouse ST2 mAb (clone 3E10) had been generated as described elsewhere [40]. FITC-conjugated and non-conjugated anti-mouse ST2 mAbs (clones DJ8 [49], [50], 245707 and 245714) were obtained from MD Bioscience and R&D Systems, respectively.

### Anti-IL-33 Abs

Anti-human/mouse IL-33 mAb (Nessy-1, Alexis), anti-mouse IL-33 mAb (518017, R&D Systems) and anti-mouse IL-33 polyAb (AF3626, R&D Systems) were used.

### Generation of anti-mouse IL-33 mAbs

Anti-mouse IL-33 mAbs were generated and provided by Medical & Biological Laboratories Co., Ltd. (Nagano, Japan). cDNA encoding the mouse IL-33 corresponding to amino acids 109–266 was expressed in *E. coli* as an N-terminal tagged fusion protein. After purification of the fusion protein, the tagged sequence was cleaved enzymatically and removed by affinity purification. Five-week-old female C3H mice (Japan SLC, Hamamatsu) were immunized with the purified protein emulsified with Freund's complete adjuvant (Sigma-Aldrich) by injection into the footpads 5 times at 1-week intervals]. Three days after the final immunization, cells from the lymph nodes of the immunized mice were fused with P3-U1 mouse myeloma cells in the presence of 50% (w/v) polyethylene glycol (PEG4000) (Wako). Hybridomas were screened by ELISA and immunoblotting to identify those generating mAbs. Positive clones were subcloned two times by limiting dilution and rescreened by ELISA and immunoblotting. The mAbs were purified from the culture supernatant using Protein A-Sepharose (GE Healthcare). The eluted antibodies were analyzed by SDS-PAGE.

### Bone marrow cell-derived and fetal liver cell-derived cultured mast cells

Mouse femoral bone marrow cell-derived cultured mast cells (BMCMCs) were generated as described elsewhere [7]. For generation of fetal liver cell-derived cultured mast cells (FLCMCs), livers were harvested from newborn TRAF6^+/+^ and TRAF6^−/−^ mice, and liver single-cell suspensions were prepared by grinding the tissues through a 70-µm nylon cell strainer (BD Falcon) with the plunger of a 5-ml disposable syringe. Bone marrow cells and fetal liver cells were cultured in the presence of 10 ng/ml rmIL-3 (PeproTech) for 6–8 weeks, at which time flow cytometry showed the cells to be a >98% c-kit^+^ FcεRIα^+^ population. Before using the cells, rmIL-3 was removed by washing. MCs (2×10^5^ cells/well in 96-well flat-bottom plates) were cultured with 1 µg/ml IgE (SPE-7, Sigma), 30 or 100 ng/ml rmIL-33 (R&D Systems) and a combination of 1 µg/ml SPE-7 plus 100 ng/ml rmIL-33 in the presence and absence of 40 or 80 µg/ml anti-mouse ST2 mAb, anti-IL-33 Ab or isotype-matched control IgG for 24 h.

### Thioglycolate (TGC)-induced macrophages

For collection of thioglycolate (TGC)-induced mouse peritoneal macrophages (TGC-macrophages), mice were injected intraperitoneally with 5 ml of 2% TGC (Nissui). Three days later, peritoneal exudate cells (PECs) were collected. TGC-macrophages (2×10^5^ cells/well in 96-well flat-bottom plates) were incubated with 0–100 ng/ml LPS (*Salmonella enterica* serotype typhimurium; SIGMA) in the presence and absence of 40 µg/ml anti-ST2 mAb, anti-IL-33 mAb or isotype-matched control IgG for 24 or 48 h.

### Flow cytometry

BMCMCs were incubated with anti-CD16/CD32 mAb (93, eBioscience; or 2.4G2, BD Biosciences) for 15 min on ice. The cells were then incubated with PE-conjugated anti-mouse FcεRIα (MAR-1, eBioscience), APC-conjugated anti-mouse c-Kit (2B8, eBioscience) and FITC-conjugated or non-conjugated anti-mouse ST2 mAb (DJ8, 3E10, 245707 or 245714) for 45 min on ice. After washing, the cells were incubated with mFITC-conjugated anti-rat IgG2b (RG7/11.1, BD Biosciences) or anti-rat IgG2a (RG7/1.30, BD Biosciences) as the second antibody for non-conjugated anti-mouse ST2 mAbs for 45 min on ice. The expression of ST2 on 7-amino actinomycin D-negative FcεRIα^+^ c-Kit^+^ BMCMCs was analyzed on a FACSCalibur flow cytometer (Becton Dickinson) using CellQuest software (Becton Dickinson).

### Cell survival

TGC-induced peritoneal macrophages (1×10^6^ cells/ml for FACS analysis and 2.5×10^5^ cells/ml for lactate dehydrogenase [LDH] release assay, respectively) were cultured in the presence and absence of 100 ng/ml LPS for 0–48 h. Cell viability was assessed using a MEBCYTO-Apoptosis kit (MBL) or LDH assay kit (CytoTox 96; Promega) as described previously [44].

### Cytokine ELISA

The levels of IL-6, IL-13 and TNF in culture supernatants were measured with mouse IL-6, IL-13 and TNF ELISA sets (eBioscience).

### ELISPOT

The number of IL-33-secreting cells by ELISPOT assay was performed as described elsewhere [44]. Breifly, MultiScreen-IP plates (MAIPS4510; Millipore) were coated with anti-mouse IL-33 polyclonal Ab (R&D Systems; 2 µg/ml in PBS) as a capture Ab at 4°C overnight. After blocking with PBS containing 10% FCS, TGC-induced peritoneal macrophages (2×10^4^/200 µl) were cultured in the presence or absence of 100 mg/ml LPS or 0.1 µg/ml PMA plus 1 µg/ml ionomycin at 37°C for 24 h or 48 h. After washing the wells, biotinylated anti-mouse/human IL-33 mAb (Nessy-1; Alexis Biochemicals, 400 ng/ml in PBS containing 10% FCS) as a detection Ab was applied and incubated at r.t. for 1 h. Then, after washing the wells, HRP-conjugated streptavidin (BD Biosciences) was added to the wells at r.t. for 1 h. AEC (Sigma) were used as substrates. Positive spots on Ab-coated plates were analyzed with NIH Image software.

### Statistics

An unpaired Student's *t*-test, 2-tailed, was used for statistical evaluation of the results.

## Results

### Effects of anti-ST2 mAbs on cytokine production by BMCMCs

Several mAbs against mouse ST2, i.e., clones DJ8 [49], [50], 3E10 [40], 245707 and 245714, have been generated to study the role(s) of ST2 in immune responses. It was recently demonstrated *in vitro* that IL-33-mediated cytokine production by macrophages was inhibited by addition of DJ8 [43], suggesting that DJ8 acts as a neutralizing Ab for IL-33 bioactivity. The crosslinking of ST2 by 3E10 enhanced Th2 cytokine production by Th2 cells *in vitro* [36], while the administration of 3E10 in mice resulted in the suppression of Th2 cell/cytokine-mediated allergic or viral airway inflammation [38], [40], [51] without depletion of ST2-expressing cells [37]. However, the effects of the other mAbs on IL-33-mediated immune cell activation remain unknown.

**Figure 1:**
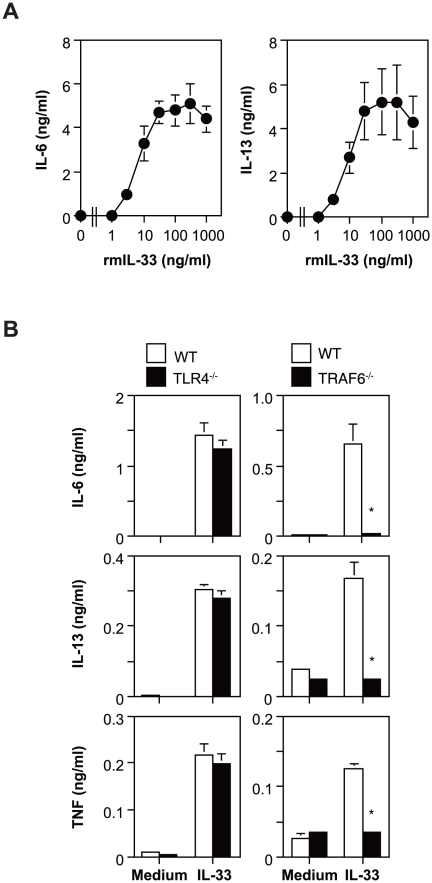
IL-33 induces TRAF6-dependent cytokine production by mast cells. BMCMCs obtained from B6J-WT mice (A) and B6J-WT and -TLR4^−/−^ mice (B; left panels) and FLCMCs obtained from B6J-WT and -TRAF6^−/−^ mice (B; right panels) were cultured in the presence of various concentration of rmIL-33 (A) or in the presence and absence of 100 ng/ml rmIL-33 for 6 h (for TNF measurement) and 24 h (for IL-6 and IL-13 measurement). The levels of IL-6, IL-13 and/or TNF in the culture supernatants were determined by ELISA. Data show the mean + SD (n = 3). *p<0.05 vs. WT.

Recombinant mouse IL-33 (rmIL-33) can induce cytokine secretion by mouse bone marrow cell-derived cultured mast cells (BMCMCs) (Fig. 1A) dependent on MyD88, which is an essential adapter molecule for signal transduction of the TLR/IL-1R (TIR) superfamily [7]. As in the case of MyD88^−/−^ BMCMCs [7] and ST2^−/−^ BMCMCs (data not shown), IL-6, IL-13 and TNF production by FLCMCs deficient in TRAF6, which is a downstream molecule of MyD88, was impaired by rmIL-33 (derived from *E. coli*) (Fig. 1B). On the other hand, rmIL-33-mediated secretion of these cytokines was observed to be comparable in wild-type (WT) and TLR4^−/−^ BMCMCs (Fig. 1), indicating that the biological activity of rmIL-33 was not influenced by contamination with endotoxin.

**Figure 2:**
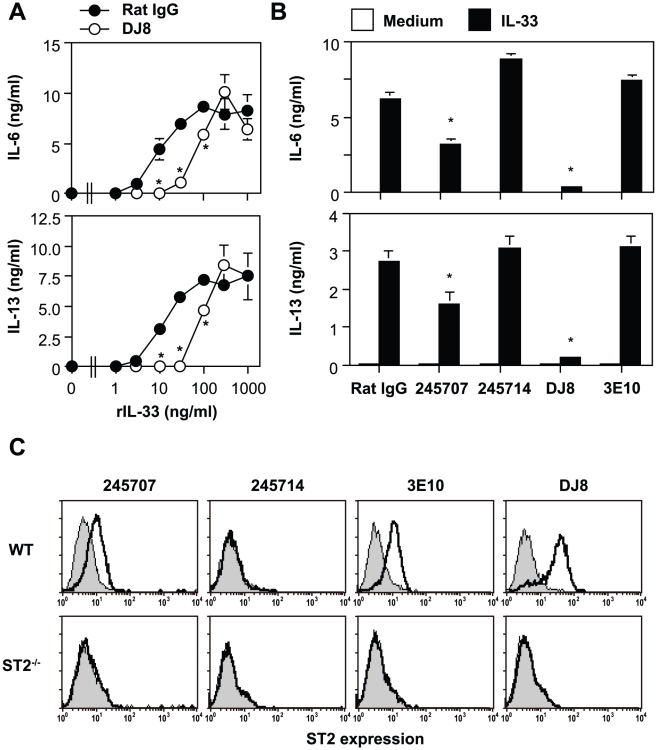
Effects of anti-ST2 mAbs on cytokine production by IL-33-stimulated BMCMCs. B6J-WT BMCMCs were stimulated with 0–1,000 ng/ml (A) or 100 ng/ml (B) rmIL-33 in the presence of 40 µg/ml of several anti-ST2 mAbs or isotype control rat IgG for 24 h. The levels of IL-6 and IL-13 in the culture supernatants were determined by ELISA. Data show the mean + SEM (n = 3). *p<0.05 vs. rat IgG+IL-33. The expression of ST2 on the cell surface of BALB-WT and ST2^−/−^ BMCMCs was determined using several distinct anti-ST2 mAb clones. Representative data by flow cytometry are shown (C). Shaded area indicates isotype-matched control IgG staining, and bold line indicates anti-ST2 mAb staining.

We next examined the effects of the anti-mouse ST2 mAbs on cytokine production by BMCMCs after IL-33 stimulation. Cytokine secretion by BMCMCs in response to 3–30 or 100 ng/mL rmIL-33 was profoundly or partially (nearly half maximum) inhibited in the presence of 40 µg/mL anti-ST2 mAb (DJ8), respectively (Fig. 2A). Therefore, we used 30 or 100 ng/mL rmIL-33 in the other neutralization studies. IL-33-mediated IL-6 and IL-13 production by WT BMCMCs was inhibited by addition of 245707 as well as DJ8, but not 3E10 or 245714 (Fig. 2B). Like rIL-33, it has been reported that crosslinking of ST2 by 3E10 promoted cytokine secretion by Th2 cells *in vitro* as an agonistic Ab [36]. On the other hand, 3E10 alone could not enhance IL-6 or IL-13 production by WT BMCMCs (Fig. 2B), although 3E10 as well as DJ8 and 245707, but not 245714, bound to ST2 on the cell surface of BMCMCs (Fig. 2C). We also found that crosslinking of ST2 by 3E10 and anti-rat IgG did not induce IL-6 or TNF production by BMCMCs (data not shown). These observations suggest that DJ8 and 245707, but not 3E10 or 245714, have neutralizing activity for IL-33-mediated mast cell activation, at least *in vitro*. Moreover, these observations indicate that the effect of 3E10 differs between Th2 cells [36] and mast cells.

### Effects of anti-IL-33 mAb on cytokine production by BMCMCs

**Figure 3:**
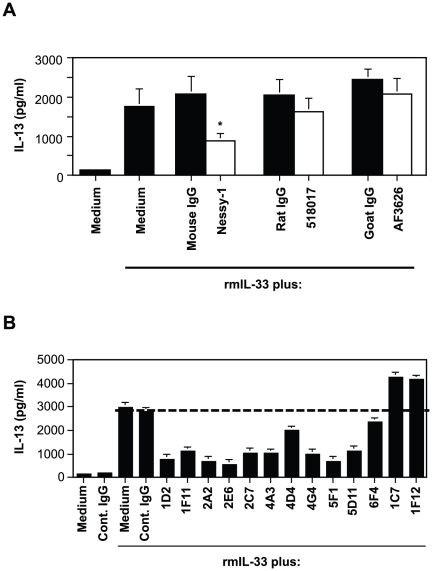
Effects of anti-IL-33 Abs on cytokine production by IL-33-stimulated BMCMCs. B6J-WT BMCMCs were stimulated with 30-ng/ml rmIL-33 in the presence and absence of commercially available anti-IL-33 Abs (A), our newly generated anti-IL-33 mAbs (B) or control IgG (A, B) for 24 h. The levels of IL-13 in the culture supernatants were determined by ELISA. Data show the mean + SEM (n = 3). *p<0.05 vs. control IgG+IL-33.

It was shown that ST2-expressing cells were depleted by anti-ST2 polyclonal Ab *in vitro* [52]. Therefore, anti-IL-33 Ab(s) rather than anti-ST2 Ab(s) would be useful for elucidating the role(s) of the IL-33-ST2 pathway *in vitro* and *in vivo*. Accordingly, we next examined the effects of anti-IL-33 mAbs (Nessy-1 and 518017) and polyclonal Ab (AF3626) on cytokine production by BMCMCs in response to rmIL-33. Nessy-1, but not 518017 or AF3626, inhibited IL-33-mediated IL-13 production by BMCMCs (Fig. 3A). However, the inhibitory effect of Nessy-1 was weak in comparison with that of the DJ8 anti-ST2 mAb, as shown in Figure 2A. Therefore, we newly generated anti-IL-33 mAbs (which were confirmed by western blot analysis to recognize rmIL-33; data not shown) and investigated their effects on IL-33-mediated cytokine production by BMCMCs. Ten (1D2, 1F11, 2A2, 2E6, 2C7, 4A3, 4D4, 4G4, 5F1 and 5D11) of 100 tested anti-IL-33 mAbs were able to inhibit IL-33-mediated IL-13 production (Fig. 3B). Like DJ8 (Fig. 2A), some of those mAbs (i.e., 2A2, 2E6 and so on) strongly inhibited IL-33 activity (Fig. 3B).

### Effects of anti-IL-33 mAbs on cytokine production by TGC-induced macrophages and BMCMCs

**Figure 4:**
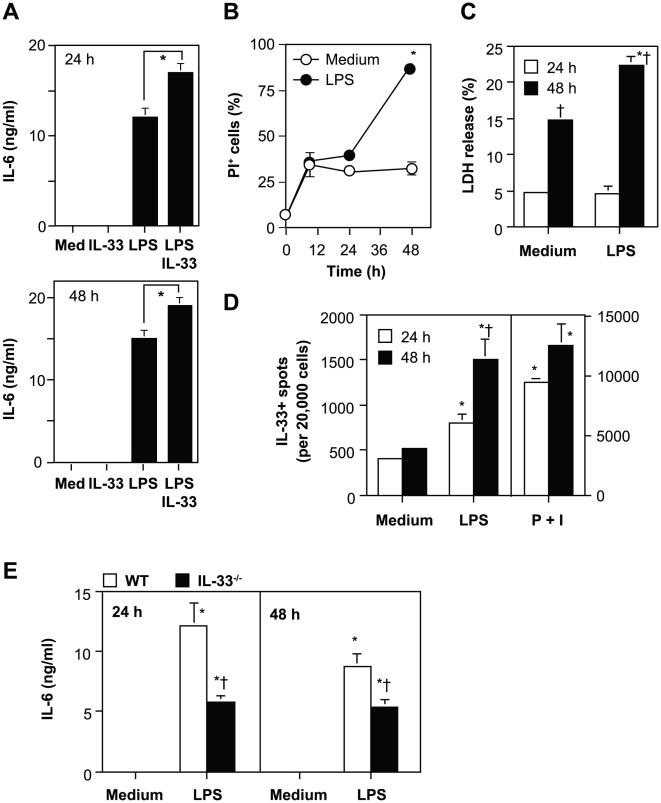
IL-33 enhances LPS-mediated cytokine production by macrophages. TGC-induced peritoneal macrophages derived from B6J-WT mice (A–D) and B6N-WT and -IL-33^−/−^ mice (E) were cultured in the presence and absence of 100 ng/ml LPS, with and without 100 ng/ml IL-33, for 9, 24 and/or 48 h. (A, E) The levels of IL-6 in the culture supernatants by ELISA. (B) The percentage of PI-positive cells by flow cytometry. (C) LDH levels in the culture supernatants. (D) The number of IL-33-secreting cells by ELISPOT. Data show the mean +/± SEM (n = 3 [A] or 4 [B–E]). *p<0.05 vs. the indicated group (A) or Medium (B–E), and ^†^p<0.05 vs. 24 h (C, D) or WT (E). P+I = PMA+ionomycin.

**Figure 5:**
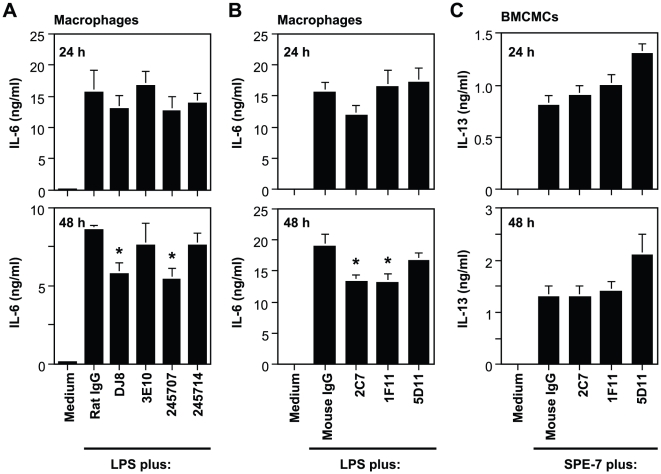
Inhibitory effects of anti-IL-33 mAbs on LPS-mediated macrophage activation by paracrine IL-33 stimulation. (A, B) TGC-induced peritoneal macrophages derived from B6J-WT mice were cultured in the presence of 100 ng/ml LPS, with and without 40 µg/ml of several anti-ST2 mAbs (A), several anti-IL-33 mAbs (B) or control IgG (A, B) for 24 and 48 h. (C) B6J-WT BMCMCs were cultured in the presence of 1 µg/ml anti-DNP IgE (SPE-7), with and without 40 µg/ml of several anti-IL-33 mAbs or control IgG for 24 and 48 h. The levels of IL-6 or IL-13 in the culture supernatants were measured by ELISA. Data show the mean + SEM ([A] n = 7, [B] n = 8 [C] n = 4). *p<0.05 vs. Rat IgG (A) or Mouse IgG (B).

It was recently reported that recombinant IL-33 enhanced LPS-mediated cytokine production by macrophages [43]. Consistent with this, we found that IL-33 augmented IL-6 production by TGC-induced peritoneal macrophages in response to LPS (Fig. 4A). We reported that TGC-induced peritoneal macrophages produced IL-33 in response to LPS [44]. In addition, it is thought that IL-33 is released by necrotic cells after stimulation [53], [54]. The proportion of annexin V-negative and propidium iodide (PI)-positive necrotic macrophages, the levels of LDH release in the culture supernatants and the number of IL-33-secreting macrophages were significantly increased at 48 h after LPS stimulation (Fig. 4B–D). Consistent with previous reports [44], we could not detect IL-33 proteins in the culture supernatants and cell lysates by ELISA and western blot analysis, respectively (data not shown). These observations suggest that necrotic macrophage-derived IL-33 may paracrinely promote cytokine production by viable macrophages after LPS stimulation. In support of this, IL-6 production by IL-33^−/−^ macrophages was reduced in comparison with WT macrophages at 24 and 48 h after LPS stimulation (Fig. 4E). To more fully elucidate this, we examined the effects of endogenous IL-33 on cytokine production by LPS-stimulated TGC-induced macrophages in the presence of anti-ST2 mAbs and anti-IL-33 mAbs. The LPS-mediated IL-6 production by TGC-induced macrophages was inhibited by addition of anti-ST2 mAbs DJ8 and 245707, but not 3E10 or 245714 at 48 h, but not 24 h, after LPS stimulation (Fig. 5A). These responses by TGC-induced macrophages were also inhibited by addition of anti-IL-33 mAbs 2C7 and 1F11, but not other mAbs including 5D11, 1D2, 2A2 and 2E6, at 48 h, but not 24 h, after LPS stimulation (Fig. 5B and data not shown). We previously demonstrated that IL-33 mRNA expression was increased in BMCMCs after stimulation with highly cytokinergic IgE [55], FcεRI-crosslinking by IgE and antigens, and PMA+ionomycin, but not LPS [44]. However, the expression level of IL-33 protein by BMCMCs was less than that by TGC-induced macrophages after stimulation [44]. In accordance with this, IL-13 production by BMCMCs was not influenced by addition of any of the anti-IL-33 mAbs at 48 h after IgE stimulation (anti-DNP IgE; SPE-7) (Fig. 5C). These observations suggest that macrophages, rather than mast cells, are potential producers of IL-33, and that macrophage-derived IL-33 can activate macrophages in a paracrine manner after LPS stimulation.

## Discussion

Like ST2^−/−^ mice [56] and mice treated with a soluble ST2-Fc fusion protein [57], mice treated with a certain anti-ST2 mAb (generated by Amgen) showed attenuated development of collagen-induced arthritis [58]. Since that ST2 mAb (Amgen) inhibited IL-33-mediated immune responses *in vitro* and *in vivo*, it is considered to act as a blocking Ab for binding of IL-33 to ST2. Conversely, mice treated with an anti-ST2 polyclonal Ab showed aggravated development of collagen-induced arthritis [52]. Since that polyclonal Ab lysed ST2-expressing cells *in vitro*, its *in vivo* administration may have depleted certain ST2-expressing regulatory cells such as Tr1 cells [59] as well as ST2-expressing effector cells such as mast cells [56], thereby causing aggravation, rather than attenuation, of the arthritis. However, the precise activities (i.e., depletion, agonism, blocking, etc.) of the other ST2 Abs were poorly characterized in the previous studies, because many of which were performed before the identification of IL-33.

It is well known that the biological activities of the IL-1 family of cytokines are elaborately regulated by decoy/soluble receptors, binding proteins and/or receptor antagonists [60], [61]. For example, the activities of IL-1α and IL-1β are mediated by IL-1R (IL-1R1 and IL-1RAcP), but blocked by IL-1R2, the soluble form IL-1Rs and IL-1 receptor antagonist (IL-1Ra) [60], [61]. The activities of IL-18 are mediated by IL-18R, but inhibited by IL-18-binding protein [60], [61]. On the other hand, inconsistent results were reported between a ligand- and its receptor-deficient mice even on the same genetic background. For example, experimental autoimmune encephalomyelitis developed normally in IL-18^−/−^ mice, but not in IL-18Rα^−/−^ mice [62]. These observations suggest involvement of another ligand(s) besides IL-18, i.e., IL-1F7 [63], in the development of the disease. Moreover, IL-1F10, in addition to IL-1α, IL-1β and IL-1Ra, also can bind to IL-1R1, although its binding affinity is low compared with IL-1β and IL-1Ra [64]. Therefore, like IL-18Rα and IL-1R1, ST2 may be a component of receptors for another ligand(s) besides IL-33. As another possibility, IL-33 may bind to other receptors besides ST2, SIGIRR/Tir8 [65] and c-Kit [66]. Thus, it was surmised that, for elucidation of the precise roles of IL-33 *in vivo* and *in vitro*, it would be more advantageous to use neutralizing Abs for IL-33 rather than for ST2. Therefore, in the present study, we newly generated anti-IL-33 mAbs and characterized their functions as well as the functions of anti-ST2 Abs.

We and the others have shown that macrophages can release IL-33 after LPS stimulation [44], and IL-33 can enhance LPS-mediated TNF and IL-6 production by macrophages (Fig. 4A) [43]. We also found that IL-33^−/−^ macrophages showed reduced IL-6 production in response to LPS (Fig. 4C). Likewise, LPS-mediated IL-6 production by macrophages was inhibited by treatment with anti-IL-33 mAbs (2C7 and 1F11, but not other mAbs) (Fig. 5B and data not shown), a soluble ST2-Fc fusion protein [15] or anti-ST2 mAbs (DJ8 and 245707, but not 3E10 and 245714) (Fig. 5A). It was reported that IL-1, IL-6, IL-12 and TNF production by macrophages from ST2^−/−^ mice on the BALB/c background was increased [16] or comparable [43] with those from wild-type mice at 12, 24 or 48 h after LPS stimulation. The apparent discrepancy between ST2^−/−^ macrophages and IL-33^−/−^/anti-ST2 mAb-treated/soluble ST2-Fc fusion protein-treated macrophages may be accounted for as described elsewhere [4]. Nonetheless, these observations (except the study using ST2^−/−^ macrophages [16]) suggest that macrophages produce IL-33 in response to LPS, and that that IL-33 then additively promotes LPS-mediated macrophage activation.

The inhibitory levels of cytokine production by macrophages treated with anti-IL-33 neutralizing Ab was lesser than those by IL-33^−/−^ macrophages after LPS stimulation. It is considered that IL-33 has dual roles as a cytokine and a nuclear factor [67], [68], [69]. The function of both secreted and nuclear IL-33 was abrogated in IL-33-deficient cells. On the other hand, the neutralizing antibody for IL-33 and/or ST2 can inhibit the effect of secreted IL-33, but not that of nuclear IL-33. Thus, the difference between anti-IL-33 neutralizing antibody-treated and IL-33-deficient macrophages may be due to the potential role of IL-33 in the nucleus.

Anti-IL-33 mAb 5D11 inhibited recombinant IL-33_109–266_-dependent IL-13 production by BMCMCs (Fig. 3B). Conversely, it did not inhibit the activity of endogenous IL-33 (probably full-length IL-33) in LPS-mediated IL-6 production by macrophages (Fig. 5B). Like anti-IL-33 mAb Nessy-1, our anti-IL-33 mAbs were generated against IL-33_109–266_, not full-length IL-33, and it seemed that some of them including 1D2, 2A2, and 2E6 could not inhibit the activities of full-length IL-33 even though they neutralized recombinant IL-33_109–266_ (Fig. 3B and data not shown).

In conclusion, our findings suggest that IL-33-neutralizing mAbs, which we newly generated, will be useful tools for the understanding the pathophysiological function(s) of IL-33 *in vitro* and presumably *in vivo*. They also have potential for aiding in the development of new therapeutics for certain IL-33-mediated disorders.
